# Supplementary material for: Contractile Work of the Soleus and Biarticular Mechanisms of the Gastrocnemii Muscles Increase the Net Ankle Mechanical Work at High Walking Speeds
Source: Biology (Basel). 2023 Jun 16;12(6):872. doi: 10.3390/biology12060872 (PMC10295290; doi:10.3390/biology12060872)
Supplement: Supplementary file 1 [file biology-12-00872-s001.zip › Supplementary_Material/Sensitivity analysis.pdf]

Table S1. Soleus (Sol) net mechanical work done at the ankle joint (first row), gastrocnemii (Gastroc) net work at the ankle (second row) and knee joint (third row) during the stance phase of walking at the investigated speeds given an EMG-activity ratio of the gastrocnemius medialis (GM) to the sum of GM and gastrocnemius lateralis EMG activity of 0.4. Variations of the Sol force-length-velocity potential (λ_{Sol}) are shown in the horizontal axes and in the gastrocnemii (λ_{Gast}) in the vertical axes. All values are averaged over fifteen participants and nine stance phases, respectively.

ratio = 0.4

Ankle net work Sol (J)

	Slow (0.7 $m.s^{-1}$)			Preferred (1.4 $m.s^{-1}$)			Transition (2.0 $m.s^{-1}$)			Maximum (2.6±0.3 $m.s^{-1}$)		
	$\lambda_{Sol}^{0.6}$	$\lambda_{Sol}^{0.7}$	$\lambda_{Sol}^{0.8}$	$\lambda_{Sol}^{0.6}$	$\lambda_{Sol}^{0.7}$	$\lambda_{Sol}^{0.8}$	$\lambda_{Sol}^{0.6}$	$\lambda_{Sol}^{0.7}$	$\lambda_{Sol}^{0.8}$	$\lambda_{Sol}^{0.6}$	$\lambda_{Sol}^{0.7}$	$\lambda_{Sol}^{0.8}$
$\lambda_{Gast}^{0.8}$	0.15	0.14	0.14	3.31	3.50	3.66	5.57	5.89	6.16	7.48	7.94	8.34
$\lambda_{Gast}^{0.7}$	0.14	0.14	0.13	3.47	3.66	3.81	5.85	6.16	6.42	7.88	8.34	8.72
$\lambda_{Gast}^{0.6}$	0.14	0.13	0.13	3.66	3.84	3.98	6.16	6.46	6.70	8.34	8.78	9.14

Ankle net work Gastroc (J)

	Slow (0.7 $m.s^{-1}$)			Preferred (1.4 $m.s^{-1}$)			Transition (2.0 $m.s^{-1}$)			Maximum (2.6±0.3 $m.s^{-1}$)		
	$\lambda_{Sol}^{0.6}$	$\lambda_{Sol}^{0.7}$	$\lambda_{Sol}^{0.8}$	$\lambda_{Sol}^{0.6}$	$\lambda_{Sol}^{0.7}$	$\lambda_{Sol}^{0.8}$	$\lambda_{Sol}^{0.6}$	$\lambda_{Sol}^{0.7}$	$\lambda_{Sol}^{0.8}$	$\lambda_{Sol}^{0.6}$	$\lambda_{Sol}^{0.7}$	$\lambda_{Sol}^{0.8}$
$\lambda_{Gast}^{0.8}$	-0.11	-0.11	-0.10	2.22	2.03	1.86	3.63	3.31	3.04	5.68	5.22	4.82
$\lambda_{Gast}^{0.7}$	-0.11	-0.10	-0.10	2.05	1.86	1.71	3.35	3.04	2.78	5.28	4.82	4.44
$\lambda_{Gast}^{0.6}$	-0.10	-0.10	-0.09	1.86	1.69	1.54	3.04	2.74	2.50	4.82	4.38	4.02

Knee net work Gastroc (J)

	Slow (0.7 $m.s^{-1}$)			Preferred (1.4 $m.s^{-1}$)			Transition (2.0 $m.s^{-1}$)			Maximum (2.6±0.3 $m.s^{-1}$)		
	$\lambda_{Sol}^{0.6}$	$\lambda_{Sol}^{0.7}$	$\lambda_{Sol}^{0.8}$	$\lambda_{Sol}^{0.6}$	$\lambda_{Sol}^{0.7}$	$\lambda_{Sol}^{0.8}$	$\lambda_{Sol}^{0.6}$	$\lambda_{Sol}^{0.7}$	$\lambda_{Sol}^{0.8}$	$\lambda_{Sol}^{0.6}$	$\lambda_{Sol}^{0.7}$	$\lambda_{Sol}^{0.8}$
$\lambda_{Gast}^{0.8}$	3.21	2.96	2.75	1.68	1.54	1.42	-0.77	-0.71	-0.65	-3.10	-2.85	-2.64
$\lambda_{Gast}^{0.7}$	2.99	2.75	2.54	1.56	1.42	1.30	-0.72	-0.65	-0.60	-2.88	-2.64	-2.43
$\lambda_{Gast}^{0.6}$	2.75	2.51	2.31	1.42	1.28	1.17	-0.65	-0.59	-0.54	-2.64	-2.40	-2.20

Table S2. Soleus (Sol) net mechanical work done at the ankle joint (first row), gastrocnemii (Gastroc) net work at the ankle (second row) and knee joint (third row) during the stance phase of walking at the investigated speeds given an EMG-activity ratio of the gastrocnemius medialis (GM) to the sum of GM and gastrocnemius lateralis EMG activity of 0.5. Variations of the Sol force-length-velocity potential (λ_{Sol}) are shown in the horizontal axes and in the gastrocnemii (λ_{Gast}) in the vertical axes. All values are averaged over fifteen participants and nine stance phases, respectively.

ratio = 0.5

		Slow (0.7 m. s^{-1})			Preferred (1.4 m. s^{-1})			Transition (2.0 m. s^{-1})			Maximum (2.6±0.3 m. s^{-1})		
		$\lambda_{Sol}^{0.6}$	$\lambda_{Sol}^{0.7}$	$\lambda_{Sol}^{0.8}$	$\lambda_{Sol}^{0.6}$	$\lambda_{Sol}^{0.7}$	$\lambda_{Sol}^{0.8}$	$\lambda_{Sol}^{0.6}$	$\lambda_{Sol}^{0.7}$	$\lambda_{Sol}^{0.8}$	$\lambda_{Sol}^{0.6}$	$\lambda_{Sol}^{0.7}$	$\lambda_{Sol}^{0.8}$
Ankle net work Sol (J)	$\lambda_{Gast}^{0.8}$	0.14	0.14	0.13	3.49	3.68	3.83	5.88	6.19	6.45	7.92	8.37	8.76
	$\lambda_{Gast}^{0.7}$	0.14	0.13	0.13	3.65	3.83	3.98	6.15	6.45	6.69	8.31	8.76	9.12
	$\lambda_{Gast}^{0.6}$	0.13	0.13	0.12	3.83	4.00	4.13	6.45	6.72	6.95	8.76	9.18	9.52
Ankle net work Gastroc (J)	$\lambda_{Gast}^{0.8}$	-0.11	-0.10	-0.10	2.03	1.85	1.69	3.32	3.01	2.76	5.24	4.78	4.40
	$\lambda_{Gast}^{0.7}$	-0.10	-0.10	-0.09	1.87	1.69	1.55	3.05	2.76	2.51	4.84	4.40	4.04
	$\lambda_{Gast}^{0.6}$	-0.10	-0.09	-0.08	1.69	1.52	1.39	2.76	2.47	2.25	4.40	3.98	3.64
Knee net work Gastroc (J)	$\lambda_{Gast}^{0.8}$	2.94	2.70	2.50	1.53	1.39	1.27	-0.71	-0.64	-0.59	-2.84	-2.60	-2.39
	$\lambda_{Gast}^{0.7}$	2.73	2.50	2.30	1.41	1.27	1.16	-0.65	-0.59	-0.54	-2.63	-2.39	-2.20
	$\lambda_{Gast}^{0.6}$	2.50	2.27	2.08	1.27	1.15	1.04	-0.59	-0.54	-0.49	-2.39	-2.17	-1.98

Table S3. Soleus (Sol) net mechanical work done at the ankle joint (first row), gastrocnemii (Gastroc) net work at the ankle (second row) and knee joint (third row) during the stance phase of walking at the investigated speeds given an EMG-activity ratio of the gastrocnemius medialis (GM) to the sum of GM and gastrocnemius lateralis EMG activity of 0.6. Variations of the Sol force-length-velocity potential (λ_{Sol}) are shown in the horizontal axes and in the gastrocnemii (λ_{Gast}) in the vertical axes. All values are averaged over fifteen participants and nine stance phases, respectively.

ratio = 0.6

Ankle net work Sol (J)

	Slow (0.7 $m.s^{-1}$)			Preferred (1.4 $m.s^{-1}$)			Transition (2.0 $m.s^{-1}$)			Maximum (2.6±0.3 $m.s^{-1}$)		
	$\lambda_{Sol}^{0.6}$	$\lambda_{Sol}^{0.7}$	$\lambda_{Sol}^{0.8}$	$\lambda_{Sol}^{0.6}$	$\lambda_{Sol}^{0.7}$	$\lambda_{Sol}^{0.8}$	$\lambda_{Sol}^{0.6}$	$\lambda_{Sol}^{0.7}$	$\lambda_{Sol}^{0.8}$	$\lambda_{Sol}^{0.6}$	$\lambda_{Sol}^{0.7}$	$\lambda_{Sol}^{0.8}$
$\lambda_{Gast}^{0.8}$	0.14	0.13	0.13	3.62	3.80	3.95	6.10	6.41	6.65	8.25	8.69	9.06
$\lambda_{Gast}^{0.7}$	0.13	0.13	0.12	3.78	3.95	4.09	6.36	6.65	6.88	8.63	9.06	9.41
$\lambda_{Gast}^{0.6}$	0.13	0.12	0.11	3.95	4.11	4.24	6.65	6.92	7.13	9.06	9.47	9.80

Ankle net work Gastroc (J)

	Slow (0.7 $m.s^{-1}$)			Preferred (1.4 $m.s^{-1}$)			Transition (2.0 $m.s^{-1}$)			Maximum (2.6±0.3 $m.s^{-1}$)		
	$\lambda_{Sol}^{0.6}$	$\lambda_{Sol}^{0.7}$	$\lambda_{Sol}^{0.8}$	$\lambda_{Sol}^{0.6}$	$\lambda_{Sol}^{0.7}$	$\lambda_{Sol}^{0.8}$	$\lambda_{Sol}^{0.6}$	$\lambda_{Sol}^{0.7}$	$\lambda_{Sol}^{0.8}$	$\lambda_{Sol}^{0.6}$	$\lambda_{Sol}^{0.7}$	$\lambda_{Sol}^{0.8}$
$\lambda_{Gast}^{0.8}$	-0.10	-0.10	-0.09	1.90	1.72	1.57	3.10	2.80	2.55	4.91	4.47	4.10
$\lambda_{Gast}^{0.7}$	-0.10	-0.09	-0.09	1.74	1.57	1.43	2.84	2.55	2.32	4.52	4.10	3.74
$\lambda_{Gast}^{0.6}$	-0.09	-0.09	-0.08	1.57	1.41	1.28	2.55	2.28	2.07	4.10	3.69	3.36

Knee net work Gastroc (J)

	Slow (0.7 $m.s^{-1}$)			Preferred (1.4 $m.s^{-1}$)			Transition (2.0 $m.s^{-1}$)			Maximum (2.6±0.3 $m.s^{-1}$)		
	$\lambda_{Sol}^{0.6}$	$\lambda_{Sol}^{0.7}$	$\lambda_{Sol}^{0.8}$	$\lambda_{Sol}^{0.6}$	$\lambda_{Sol}^{0.7}$	$\lambda_{Sol}^{0.8}$	$\lambda_{Sol}^{0.6}$	$\lambda_{Sol}^{0.7}$	$\lambda_{Sol}^{0.8}$	$\lambda_{Sol}^{0.6}$	$\lambda_{Sol}^{0.7}$	$\lambda_{Sol}^{0.8}$
$\lambda_{Gast}^{0.8}$	2.75	2.51	2.31	1.42	1.28	1.17	-0.66	-0.60	-0.55	-2.65	-2.41	-2.21
$\lambda_{Gast}^{0.7}$	2.54	2.31	2.12	1.30	1.17	1.07	-0.61	-0.55	-0.50	-2.44	-2.21	-2.02
$\lambda_{Gast}^{0.6}$	2.31	2.09	1.91	1.17	1.05	0.95	-0.55	-0.49	-0.45	-2.21	-2.00	-1.82

Table S4. Soleus (Sol) net mechanical work done at the ankle joint (first row), gastrocnemii (Gastroc) net work at the ankle (second row) and knee joint (third row) during the stance phase of walking at the investigated speeds given an EMG-activity ratio of the gastrocnemius medialis (GM) to the sum of GM and gastrocnemius lateralis EMG activity of 0.7. Variations of the Sol force-length-velocity potential (λ_{Sol}) are shown in the horizontal axes and in the gastrocnemii (λ_{Gast}) in the vertical axes. All values are averaged over fifteen participants and nine stance phases, respectively.

ratio = 0.7

		Slow (0.7 $m.s^{-1}$)			Preferred (1.4 $m.s^{-1}$)			Transition (2.0 $m.s^{-1}$)			Maximum (2.6±0.3 $m.s^{-1}$)		
		$\lambda_{Sol}^{0.6}$	$\lambda_{Sol}^{0.7}$	$\lambda_{Sol}^{0.8}$	$\lambda_{Sol}^{0.6}$	$\lambda_{Sol}^{0.7}$	$\lambda_{Sol}^{0.8}$	$\lambda_{Sol}^{0.6}$	$\lambda_{Sol}^{0.7}$	$\lambda_{Sol}^{0.8}$	$\lambda_{Sol}^{0.6}$	$\lambda_{Sol}^{0.7}$	$\lambda_{Sol}^{0.8}$
Ankle net work Sol (J)	$\lambda_{Gast}^{0.8}$	0.13	0.13	0.12	3.73	3.90	4.04	6.28	6.57	6.80	8.50	8.93	9.29
	$\lambda_{Gast}^{0.7}$	0.13	0.12	0.12	3.88	4.04	4.18	6.53	6.80	7.03	8.88	9.29	9.64
	$\lambda_{Gast}^{0.6}$	0.12	0.12	0.11	4.04	4.20	4.33	6.80	7.06	7.27	9.29	9.69	10.00
Ankle net work Gastroc (J)	$\lambda_{Gast}^{0.8}$	-0.10	-0.09	-0.09	1.80	1.62	1.48	2.92	2.63	2.40	4.65	4.22	3.86
	$\lambda_{Gast}^{0.7}$	-0.10	-0.09	-0.08	1.64	1.48	1.34	2.67	2.40	2.17	4.28	3.86	3.52
	$\lambda_{Gast}^{0.6}$	-0.09	-0.08	-0.08	1.48	1.32	1.20	2.40	2.14	1.93	3.86	3.47	3.15
Knee net work Gastroc (J)	$\lambda_{Gast}^{0.8}$	2.59	2.36	2.17	1.33	1.20	1.09	-0.62	-0.56	-0.51	-2.49	-2.27	-2.08
	$\lambda_{Gast}^{0.7}$	2.39	2.17	1.99	1.22	1.09	0.99	-0.57	-0.51	-0.47	-2.30	-2.08	-1.89
	$\lambda_{Gast}^{0.6}$	2.17	1.96	1.78	1.09	0.98	0.89	-0.51	-0.46	-0.42	-2.08	-1.87	-1.70
